# The Influence of Dental Disease upon Lactation

**Published:** 1917-04

**Authors:** 


### THE INFLUENCE OF DENTAL DISEASE UPON LACTATION

---

The general lowering of health which may follow various forms of dental disease, particularly those associated with a more or less severe degree of sepsis of the mouth, has come to be fully recognized by physicians; nevertheless we doubt whether all possible inferences and lessons have been drawn from this important knowledge. It must be admitted that the influence of septic conditions of the mouth is a factor deserving attention when met with in nursing women; and for this reason a contribution on this subject by Dr. Harold Waller to *The Lancet* (November 4, 1916) is of interest.

It would be an error to suppose that dental disease, although it militates against successful breast feeding, is responsible for all instances in which children fail to thrive on breast milk. Quite often failure of lactation may be the result of a variety of other causes and factors, and it is for the physician to ascertain these. However, in numerous instances in which the child is subject to intractable vomiting either during or shortly after nursing, in which sleep is disturbed and attacks of screaming are frequent, and in which the gain of the baby is unsatisfactory, the author has learned to attach importance to the presence of oral sepsis in the mother, although he finds that mere questioning about the condition of the teeth seldom affords enlightenment. If however, the inquiry is directed to the metastatic effects of oral sepsis, positive evidence will be given unsuspectingly.

Thus, it is common to be told that the woman in question is rheumatic and is subject to recurrent sore throat; that ''her head troubles her'' or that she always wakes in the morning with a bad taste in the mouth. Attacks of neuralgia, stiff neck, swollen face or ''gumboils'' are sometimes admitted. Indigestion, vomiting, loss of appetite, and a decline in weight and strength are symptoms often mentioned spontaneously.

Examination of the mouth suggests most strongly the probable etiology of many of these complaints. A superficial inspection often makes it obvious that good general health is not possible with the conditions there found. Caries of long standing, dead teeth, broken roots regarded as harmless and often covered by plates, the openings of sinuses from which pus can be expressed in quantity, loose teeth and a copious discharge arising from their alveolar sockets—all these are of common occurrence. A chronically infected state of the tonsils must necessarily be added to the list. It is, unfortunately, common to hear that such patients have for years relied on medicinal remedies for their debility and anemia.

Presenting greater difficulty, but often of equal importance, are the cases where elaborate architectural devices have been built up and where the mouth contains a number of gold-crowned teeth; for, despite their cleanly appearance, they do not always, as a matter of fact, rest upon sound foundations. The very form of their construction sometimes serves to imprison disease that has progressed beneath. So, also, may the costliness of their erection prove a serious hindrance to their alteration or removal. In such cases, the knowledge of an expert and impartial dentist may have to be supplemented by skiagrams before the point can be settled.

In contrast to the frequency with which internal disturbances in nursing babies may be traced to existence of dental disease in the mother, Dr. Waller lays stress upon the fact that digestive disturbances and intestinal disorders are very infrequent in breast-fed infants whose mothers have perfect teeth.

If the removal of dental disease is carried out adequately, even if this has given rise to a severe disturbance both in mother and child, improvement in the mother's health is sufficiently rapid and substantial to be of signal benefit to her babe. In order to definitely establish the causal relation, the author proposes as criterion that, after proper treatment of the dental disease, the rate of gain in the child's weight should be accelerated and the length of time over which nursing can be carried out should be prolonged. Further, the mother should become aware of an increase in the flow of milk.

How interest in these focal infections, so called, is growing! No physician called upon to treat a case of rheumatism, arthritis deformans, myalgia, neuritis, endocarditis, or other painful disease of obscure origin can now afford to neglect the careful physical examination so long insisted upon—too often academically—by the great clinicians. It is encouraging that we are now looking more closely for *causes*—encouraging, also, that this search is giving us a more satisfactory therapy.—*Clinical Medicine.*

